# Supplementary material for: Stable Sparse Classifiers predict cognitive impairment from gait patterns
Source: Front Psychol. 2022 Aug 16;13:894576. doi: 10.3389/fpsyg.2022.894576 (PMC9425080; doi:10.3389/fpsyg.2022.894576)
Supplement: Supplementary file 1 [file Data_Sheet_1.PDF]

Supplementary Material

1 Supplementary Figures and Tables

1.1 Supplementary Figures

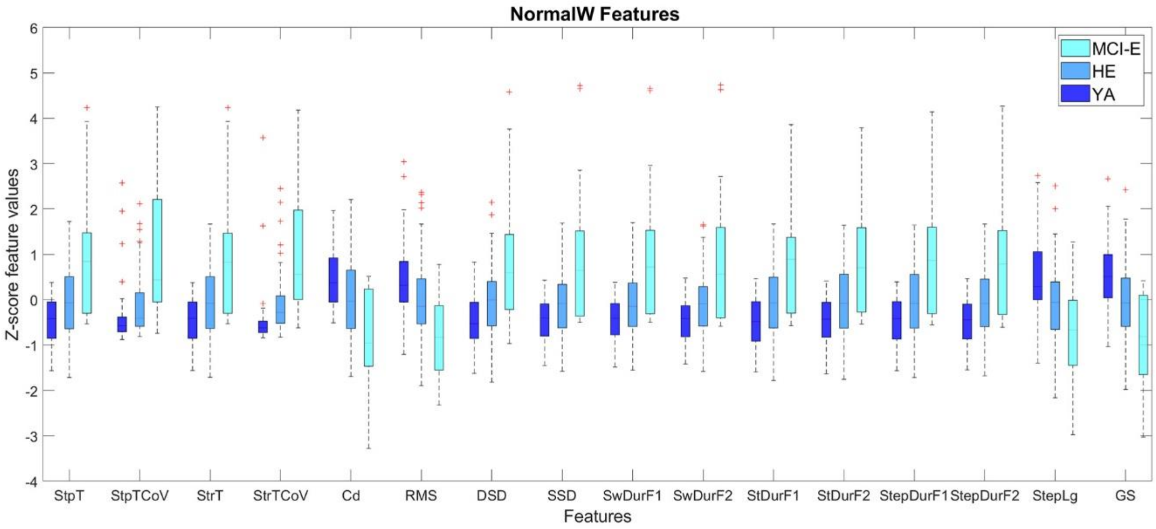

Supplementary Figure 1. Z-scores of STGF for the three groups in NormalW walking task.

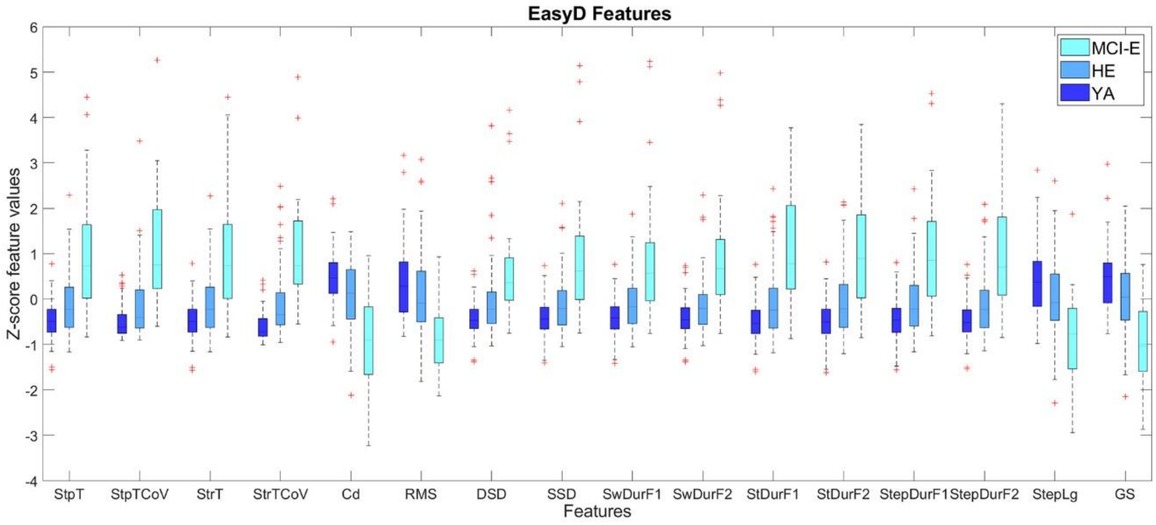

Supplementary Figure 2. Z-scores of STGF for the three groups in EasyD walking task.

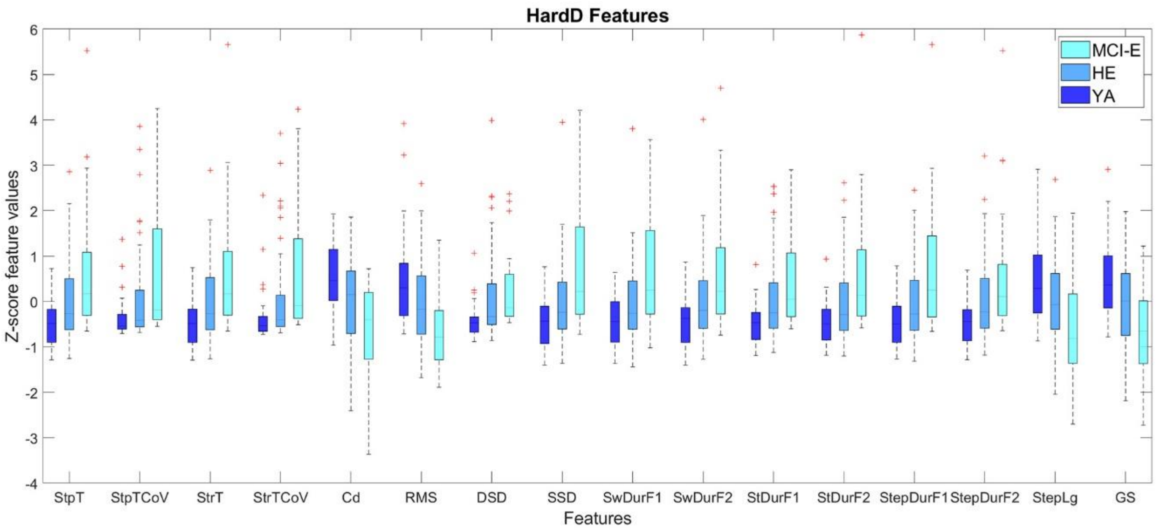

Supplementary Figure 3. Z-scores of STGF for the three groups in HardD walking task.

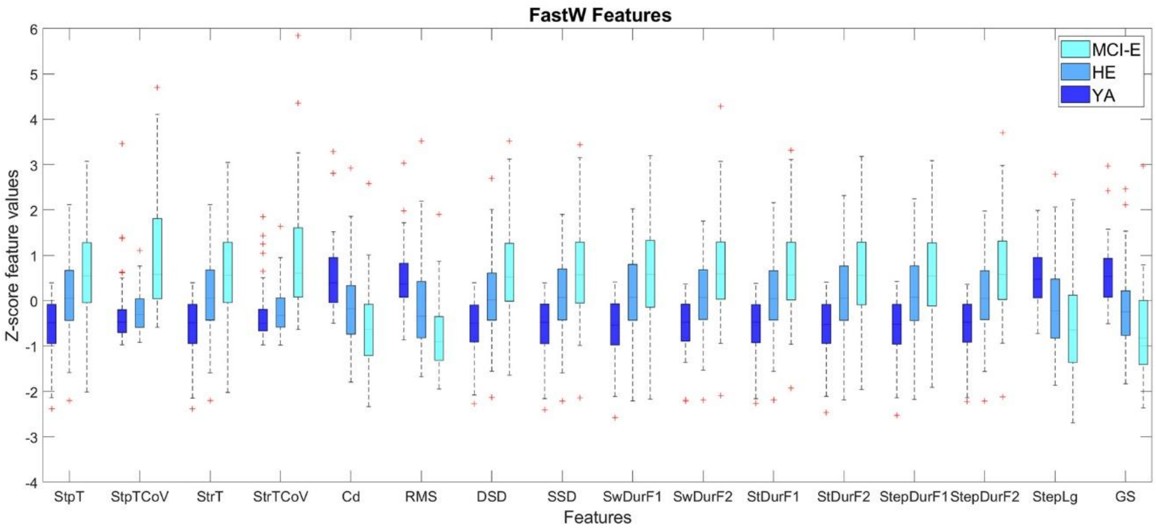

Supplementary Figure 4. Z-scores of STGF for the three groups in FastW walking task.

1.2 Supplementary Tables

Supplementary Table 1. Results of the non-parametric permutation two-way analysis of variance test for each STGF, with GROUP and TASK as main effects, and the interaction between them.

	F(2,488) group	p group	F (3,488) task	p task	F(6,488) interaction	p interaction
**StpT**	60.60	0.00	67.00	0.00	1.78	0.17
**StpTCoV**	28.78	0.00	30.88	0.00	3.33	0.02
**StrT**	60.85	0.00	67.27	0.00	1.76	0.15
**StrTCoV**	29.87	0.00	29.53	0.00	2.53	0.05
**Cd**	61.58	0.00	91.70	0.00	0.83	0.47
**RMS**	50.90	0.00	84.75	0.00	2.27	0.07
**DSD**	26.83	0.00	42.32	0.00	2.22	0.08
**SSD**	63.90	0.00	62.35	0.00	1.67	0.16
**SwDurF1**	63.57	0.00	63.66	0.00	1.60	0.20
**SwDurF2**	61.64	0.00	58.77	0.00	1.72	0.15
**StDurF1**	52.42	0.00	61.55	0.00	1.92	0.13
**StDurF2**	54.22	0.00	65.73	0.00	1.81	0.13
**StepDurF1**	60.90	0.00	68.34	0.00	1.64	0.17
**StepDurF2**	57.78	0.00	62.98	0.00	1.82	0.15
**StepLg**	50.92	0.00	62.49	0.00	0.67	0.59
**GS**	63.55	0.00	99.00	0.00	1.12	0.33
